# An efficient approach to study membrane nano-inclusions: from the complex biological world to a simple representation

**DOI:** 10.1039/d1ra00632k

**Published:** 2021-03-16

**Authors:** M. Lemaalem, N. Hadrioui, S. El Fassi, A. Derouiche, H. Ridouane

**Affiliations:** Laboratoire de Physique des Polymères et Phénomènes Critiques, Sciences Faculty Ben M'Sik, Hassan II University P.O. Box 7955 Casablanca Morocco mohammedlemaalem@gmail.com

## Abstract

Membrane nano-inclusions (NIs) are of great interest in biophysics, materials science, nanotechnology, and medicine. We hypothesized that the NIs within a biological membrane bilayer interact *via* a simple and efficient interaction potential, inspired by previous experimental and theoretical work. This interaction implicitly treats the membrane lipids but takes into account its effect on the NIs micro-arrangement. Thus, the study of the NIs is simplified to a two-dimensional colloidal system with implicit solvent. We calculated the structural properties from Molecular Dynamics simulations (MD), and we developed a Scaling Theory to discuss their behavior. We determined the thermal properties through potential energy per NI and pressure, and we discussed their variation as a function of the NIs number density. We performed a detailed study of the NIs dynamics using two approaches, MD simulations, and Dynamics Theory. We identified two characteristic values of number density, namely a critical number density *n*_c_ = 3.67 × 10^−3^ Å^−2^ corresponded to the apparition of chain-like structures along with the liquid dispersed structure and the gelation number density *n*_g_ = 8.40 × 10^−3^ Å^−2^ corresponded to the jamming state. We showed that the aggregation structure of NIs is of fractal dimension *d*_F_ < 2. Also, we identified three diffusion regimes of membrane NIs, namely, normal for *n* < *n*_c_, subdiffusive for *n*_c_ ≤ *n* < *n*_g_, and blocked for *n* ≥ *n*_g_. Thus, this paper proposes a simple and effective approach for studying the physical properties of membrane NIs. In particular, our results identify scaling exponents related to the microstructure and dynamics of membrane NIs.

## Abstract 2

Membrane nano-inclusions are of great interest in biophysics, materials science, nanotechnology, and medicine. In this work, We combined MD simulations and theories to reveal their physics behavior.

## Graphical abstract

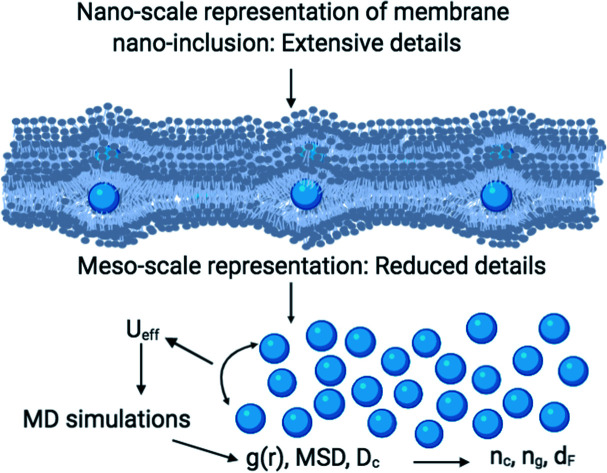

## 1 Introduction

A lipid bilayer is a thin polar membrane composed of two layers of lipid molecules. These membranes form a continuous barrier around cells and are essential for their homeostasis by regulating the ions' and molecules' diffusion through them.^1,2^ The cell membrane of almost all living organisms and many viruses are made of a lipid bilayer,^3^ just like the membranes surrounding the cell nucleus and organelles. The lipid bilayers are impermeable to hydrophilic molecules and particularly to ions. This selectivity enables the cells to regulate, in particular, the pH and salinity of their cytosol by using transmembrane proteins. Thus, provide a transporter function capable of generating and maintaining a concentration gradient and the extracellular environment.^4^ Biological membranes usually contain other constitutions than phospholipids. These proteins and other integrated components are involved in several pathologies, including cancer.^5^ In this context, W. Yang *et al.* reported a mechanism by which the anti-tumor response of cells used in cancer immunotherapy can be potentiated by modulating cholesterol metabolism.^6^ The cholesterol level in cell membranes determines the physicochemical properties of these membranes.^7^ In addition to these organic constituents, the membrane may contain inorganic nano-inclusions (NIs), such as small and macro-ions, or more complex structures that can play important roles. In this context, A. O. Elzoghby *et al.* show that protein–inorganic hybrid nano-inclusions offer promising opportunities for drug delivery and tissue imaging, particularly in the field of oncology.^8^ Besides, they are the lifeblood in thin-film composite membranes used in desalination.^9^

NIs impose a well-defined membrane deformation is the subject of intensive studies due to the potential opportunities that they offer for biomedical applications in diagnosis and therapy because of their rigidity and their perfect monodispersed character.^10–15^

To our knowledge, the techniques for determining the physical properties of nano-inclusions remain rare and still to this day a promising field to explore, in particular using MD simulation. However, many theoretical and experimental studies have been interested in determining the interactions between NIs across the membrane.^15–20^ In this context, I. Koltover and coworkers presented experimental studies of the aggregation of spherical colloidal particles adhering to lipid membranes.^18^ They observed that two-particles aggregation was found consistent with a short-range-attractive interaction and no obtained sign of a long-range one. Surprisingly, triplets of particles form chains, and a ring-shaped aggregate is observed around the vesicle size. Also, linear chain arrangements are obtained in simulations of similar situations for some particle size and adhesion regimes,^19,20^ using a scaling argument to show that this was not due to membrane-mediated interactions.^20^ But it is due to the adhesion of the particles to the membrane since a linear aggregate gives a higher adhesion area than a compact one. Therefore, such a phenomenon would not occur in the case of inclusions.^16^ Costanza Montis *et al.* performed several experiments to compare the interaction of Extracellular Vesicles-derived (EVSLB) and synthetic Supported Lipid Bilayers (SLBs) with cationic superparamagnetic iron oxide nanoparticles (SPIONs). Their experimental results revealed that the SPIONs-EVSLBs interaction is significant in comparison to SPIONs-SLBs.^17^

Particles that form jammed structures are ubiquitous in nature and omnipresent in many consumer products.^21^ Being metastable solids, they are mechanically rigid structures that generally form at low particle density due to a spatial cluster of aggregated particles. The colloid aggregation is controlled by the number density, the attractive interaction potential, and the temperature.^22^

Colloid aggregations in spanning network systems are known as gelation or coagulation.^23,24^ Thus, several works have been devoted to determinating the physical parameters and demonstrating phase boundary mapping of the phase transition by examining a range of volume fractions, temperature, and interaction strength between particles.^25–35^

**Fig. 1:**
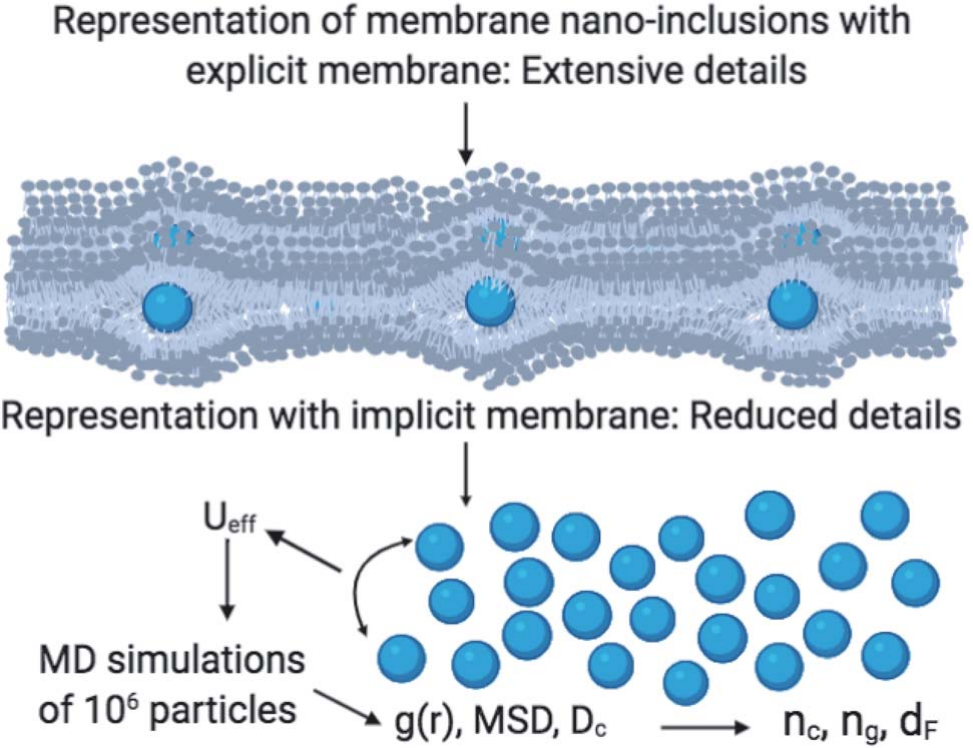
A schematic representation summarizes the research design. In the MD simulations, we considered the membrane as a two-dimensional implicit liquid for the sake of simplicity. Thus the nano-inclusions are treated as a two-dimensional colloidal suspension interacting *via* an efficient interaction *U*_eff_. Then, these considerations reduce several details and then allow the study of a large number of nano-inclusions. The numerical data of the radial-distribution-function *g*(*r*), the mean-square-displacement MSD, and the diffusion coefficient *D*_c_ allow the determination of the gelation number density *n*_g_, the fractal dimension *d*_F_, and other related parameters.

As schematically shown at the top of Fig. 1, membrane nano inclusions interact with the surrounding membrane lipids. Also, they interact with other inclusions. Furthermore, they can form complexes with other membranes NIs and with integral proteins. Thus, an all-atom MD simulation has many details that cost a lot of time in computing and programming skills. A simple alternative is to determine the direct interaction between the particles under study, where the other particles are eliminated and replaced by their effects. These effects appear in the expression of the simplified interaction potential with a relatively simple mathematical formula. From a mesoscopic viewpoint, we consider the membrane constituents as a two-dimensional homogeneous fluid since these biological constituents have almost the same structural and dynamics properties.^36^ In this case, we simplify the study of the structure and dynamics of NIs into a two-dimensional colloidal system with an implicit solvent (membrane). D. Constantin and coworkers, in 2008, determined the direct interaction potential between BuSn12 NIs inserted within diluted surfactant bilayers by using X-ray synchrotron scattering.^15,37^ Nine years later, M. Benhamou and coworkers added a prominent correction to describe the interaction between NIs within biological membrane bilayer.^38^ In the work of Constantin *et al.*,^15,37^ the repulsive interaction between inorganic BuSn12 nanoparticles inserted into surfactant bilayers was measured using Small X-ray Synchrotron Scattering. Accordingly, the analysis of the measured structure factor of the two-dimensional fluid formed by the inserted nanoparticles led to their membrane-mediated interaction. This interaction is repulsive and well fitted to a Gaussian function. Thus, M. Benhamou *et al.*^38^ have added an attractive term to the repulsive interaction form proposed by D. Constantin. This form can describe will the NIs interaction within a biological membrane bilayer.

In this case, we simplify the study of the structure and dynamics of NIs into a two-dimensional colloidal system with an implicit solvent (membrane). From previous works, D. Constantin and coworkers, in 2008, determined the direct interaction potential between BuSn12 NIs inserted within diluted surfactant bilayers by using X-ray synchrotron scattering.^15,37^ Nine years later, M. Benhamou and coworkers added a prominent correction to describe the interaction between NIs within biological membrane bilayer.^38^ In the work of Constantin *et al.*,^15,37^ the repulsive interaction between inorganic BuSn12 nanoparticles inserted into surfactant bilayers was measured using Small X-ray Synchrotron Scattering. Accordingly, the analysis of the measured structure factor of the two-dimensional fluid formed by the inserted nanoparticles led to their membrane-mediated interaction. This interaction is repulsive and well fitted to a Gaussian function. Thus, M. Benhamou *et al.*^38^ have added an attractive term to the repulsive interaction form proposed by D. Constantin. This form can describe will the NIs interaction within a biological membrane bilayer.

In this work, we expect the NIs to undergo a phase transition, corresponding to the critical and the gelation number-densities, that we note *n*_c_ and *n*_g_, respectively. For these densities, we expect the physical properties to present a discontinuity. For examining this hypothesis, we apply MD simulations, Scaling Theory, and Dynamics Theory to investigate the structure and dynamics of NIs in bilayer membranes. For this, we shall perform a series of simulations for various number densities. Thus, we determine *n*_c_ and *n*_g_, and we quantify the relevant scaling exponents. For this purpose, the structure and dynamics behavior will be discussed using a detailed analysis of the radial distribution function and the mean square displacement. This paper proposes a simple and effective approach for detecting the gelation phenomenon, relevant for many aggregation processes at the level of biological membranes. In particular, our results identify scaling exponents related to the microstructure and dynamics of membrane NIs.

## 2 Simulation method

### 2.1 Interaction potential

In this work, we considered an assembly of monodisperse spherical inorganic nanoparticles immersed in a two-dimensional bilayer-membrane. We assumed that these nano-inclusions are rigid and charge-neutral. We denoted by 
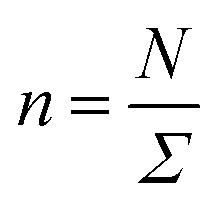
 and *d* = 2*R*, the nanoparticle number density, and their common diameter, respectively. *N* accounts for their number, *Σ* for the membrane area, and *R* stands for their radius. Because of the membrane undulations, the immersed nanoparticles experience a membrane-mediated repulsive force. The latter was measured using Synchrotron Small-Angle X-ray Scattering.^15,37^ The interaction potential determined with the help of the structure factor measured using RPA.^39^ The latter compared to the theoretical one, which explicitly contains the pair-potential. The reference was the structure factor of a hard-core disc model. Herein, we recall simply the expression of the membrane-mediated repulsive potential,1
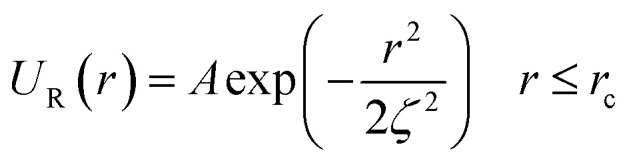
where *U*_R_(*r*) represents the repulsive part of the total interaction potential, and *r* is the separation distance between the particles. The potential amplitude *A*, whose magnitude is in the order of some thermal energies *k*_B_*T*. *r*_c_ is a cutoff distance, for which the interaction potential tends to zero, used in the MD simulations to reduce the calculation time, and *ζ* represents the potential range. We give an explication to this distance-scale by saying that it presents the length of the correlation in the plane (size of the hole and the valley of membrane's undulations). The latter depends on the bilayer membrane characteristics^40–42^ through the modulus of curvature, *κ*, and the interfacial tension, *γ*.

According to M. Benhamou *et al.* corrections,^38^ the characteristics of the membrane *κ*, *γ*, the potential amplitude *A*(*n*) and the renormalized range *ζ*(*n*) depend on the number density of NIs, *n*. Thus, to propose an explicit variation-law to present these quantities as a function of the density, the authors assert it is a typical value, *n*_0_, where the bilayer membrane ceases to be fluid, then becomes rigid. Therefore, at *n* = *n*_0_, the ripple of the membrane disappears, that is, *ζ*(*n*) = 0. This implies that *A*(*n*) = 0. Also, the authors say that the special value *n*_0_ corresponds to the situation where the nanoparticles are extremely congested, then cover the entire surface of the membrane. Thus, *n*_0_ is defined as follows: 
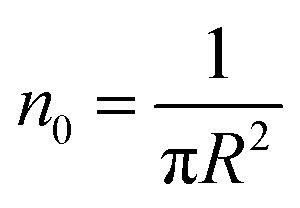
, where π*R*^2^ is the disc surface. Thus, they assume, intuitively, that the amplitude *A*(*n*) and the range *ζ*(*n*) must decrease when the density *n* increases. For these quantities, they expect the following approximate forms,2
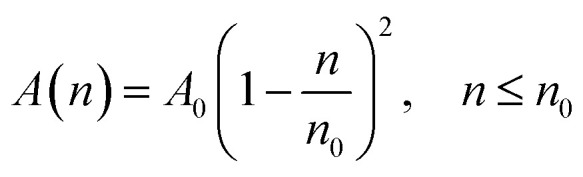
3
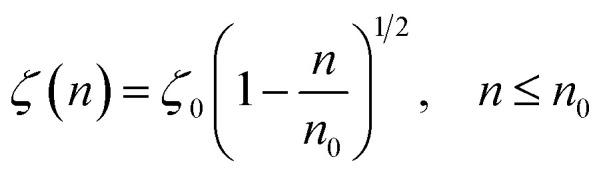
here, *ζ*_0_ denotes the bulk value of the in-plane correlation length (in the absence of nanoparticles). The constant *A*_0_ that measures the number of contacts between interacting NIs should be proportional to the inverse of the squared bending modulus *κ*,^43–45^ that is *A*_0_ ∼ *κ*^−2^, where the proportionality coefficient is known^43–45^ and presents as a combination of the thermal energy *k*_B_*T* and the nanoparticle diameter, *d*. Therefore, as it should be, the latter vanishes for the rigid bilayer-membranes *κ* → ∞.

Besides the above repulsive potential, the NIs experience naturally an attractive force of van der Waals type,^46^4
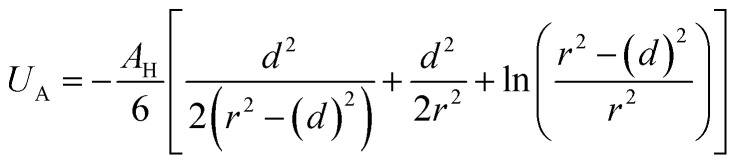
whose corresponding potential reads (in Derjaguin approximation^47^),5
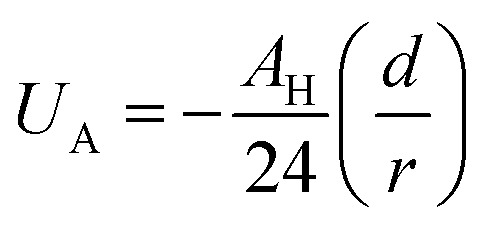
where *d* = 2*R* is the NIs diameter and *A*_H_ denotes the Hamaker constant, which is in the 10^−22^ to 10^−19^ J range. Therefore, the overall effective potential *U*_eff_(*r*) is,6
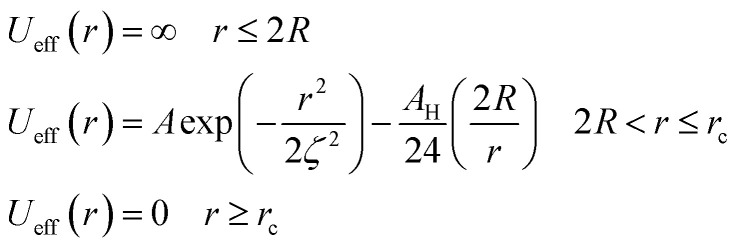


Thus, the pair interaction potential between membrane NIs is the sum of a repulsive term, originating from the bilayer-membrane undulations, and an attractive one derived from the polarity of the host medium. We note that, in contrast to the repulsion, the VDW attraction is negligible for low-density NIs, but it is indispensable for high-density NIs.

### 2.2 Simulation details

In this work, we discuss a particular type of spherical NIs, namely the BuSn12 nanoparticles. Where BuSn12 denotes {(BuSn)_12_O_14_(OH)_6_}^2+^(4-CH_3_C_6_H_4_SO_3_^−^)_2_, the butyltin oxo cluster. The synthesis and characterization of BuSn12 is described in detail by Eychenne-Baron *et al.*^48^ BuSn12 inclusions are considered as a spherical nanoparticles of radius *R* = 4.5 Å and molecular weight of 2866.7. The corresponding parameters of the effective interaction potential, discussed above, are as follows: *n*_0_ = 15.72 × 10^−3^ Å^−2^, *A*_0_ = 41.1 × 10^−19^ J, *ζ*_0_ = 14.4 Å, and *A*_H_ = 1.5 × 10^−19^ J.

In the come after, and for a purpose to optimize the calculation time, we use the dimensionless unit, meaning that all quantities are unitless in the MD simulations.^49,50^ Accordingly, the distance is reduced by the NIs radius *R*, the number density is reduced by *R*^−2^ the energy is reduced by *k*_B_*T*, the temperature is reduced by the room temperature *T*_a_ = 298.15 K, the time is reduced by 
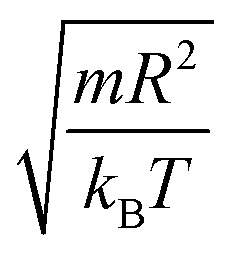
, and the pressure is reduced by 
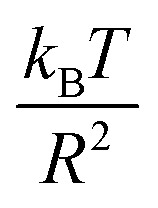
. In this work, we calculated all dynamics and structural properties using *NΣT* MD simulations by analyzing the trajectory of each particle. The simulated system consists of 10^6^ colloidal particles in a square lattice, with applied periodic boundary conditions in the two dimensions. The *NΣT* statistical ensemble conditions are adjusted using temporal integration on non-Hamiltonian Nose–Hoover motion equations. These equations generate the positions and the velocities sampled from the canonical ensemble (*NΣT*). Thus, adapt these kinetic quantities of NIs at each time step. This style fixes the temperature by adding dynamics variables coupled with particle velocities. The equations of motion used are those of Shinoda *et al.*,^51^ which associate the hydrostatic equations of Martyna *et al.*^52^ with the strain energy proposed by Parrinello and Rahman.^53^ Temporal integration schemes closely follow the Verlet integrators, preserving the measures and reversible in time, derived from Tuckerman *et al.*^54^ We performed the MD simulations using the LAMMPS software package.^49^ We started from a random initial configuration. Then, the initial configuration is equilibrated in the *NVT* ensemble before the production of the results. In this way, any proposed initial arrangement leads to the same equilibrium. We run the simulations at different densities above and beyond the gelation density 
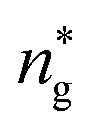
. The system is balanced long enough (1 000 000 time-step) with a time-step Δt* = 0.01.

To analytically determine *n*_c_ and *n*_g_, we go back to the effective interaction potential. We note that, in addition to the number density, there are other relevant parameters that we have considered fixed, namely the temperature and the NIs size. Practically, we characterized *U*_eff_ by two behaviours, attractive or repulsive, in other words, negative or positive. From the literature, M. Benhamou *et al.* found that *n*_c_ is unique and given by:^38^7
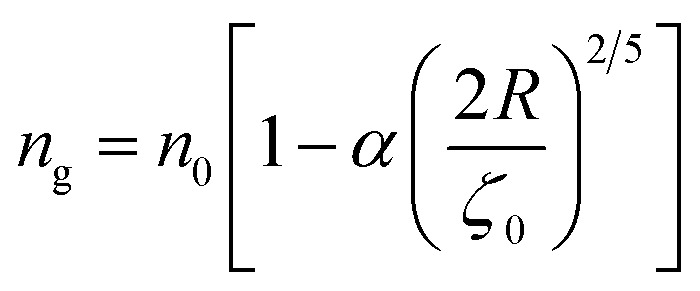
with *α* a dimensionless coefficient8
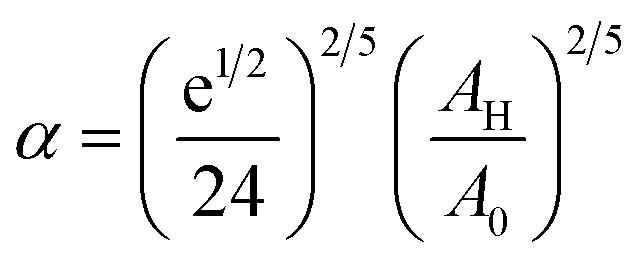
The critical number density, *n*_c_, is of the order of *n*_c_ = 3.6 × 10^−3^ Å^−2^, for BuSn12 NIs with the effective interaction potential parameters:^38^*A*_H_ = 1.5 × 10^−21^ J, *A*_0_ = 41.1 × 10^−21^ J, *ζ*_0_ = 14.4 Å, and *R* = 4.5 Å.

From the theoretical investigation, we summarize the following number density regimes comparing the number density *n* with the predicted threshold of *n*_c_:

(1) Low-density regime corresponding to *n* < *n*_c_: in this regime, the potential exhibits two zeros and two extremums, with an energy barrier between the two zeros. The first extremum is a minimum around which the immersed nanoparticles flocculate. Therefore, in the low-density regime, the potential presents a high energy barrier that prevents NIs gelation.

(2) First critical density regime corresponding to *n* ≈ *n*_c_: for this critical value, the potential is always attractive and exhibits one maximum, which is a zero at the same time for *r* = *ζ*(*n*_c_) and a secondary minimum at the right. At this specific value of density, the energy barrier disappears.

(3) High-density regime corresponding to *n* > *n*_c_: in this regime, the potential is purely attractive and presents as an increasing monotone function of the surface-distance *r*. This is the region of a prevised gelation number density *n*_g_. The first gelation number density will be determined using MD simulation.

## 3 Results and discussions

### 3.1 Structural properties

The structural analysis has been conducted using the two-dimensional Radial Distribution Function (RDF) and Coordination Number (*N*_c_).

The RDF is defined as the probability of finding two NIs at a distance *r*, each to other.9
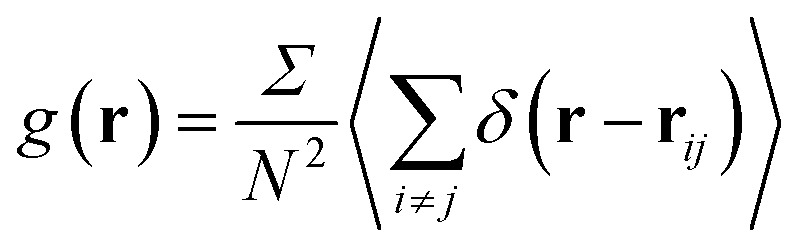
where *N* is the number of NIs and *Σ* is the area of the host membrane. In the MD framework, we performed the above sum over all pairs of nano-inclusions.

The average density distribution of NIs, at any point, is referred to as the bulk-density, *n*_b_. Thus, the average density of the NIs at a given distance, *r*, from the center of any considered NIs, *n*_r_, is related to RDF by:

10 *n*_r_ = *n*_b_*g*(*r*)

We define the coordination number *N*_c_ as the number of neighbors NIs that are within the specified cutoff distance of the pair interaction potential from a central inclusion,11
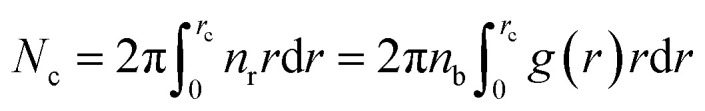
where *r*_c_ stands for the position in which the interaction potential between NIs goes to zero. Thus, the running coordination number, *N*(*r*), is defined by,12
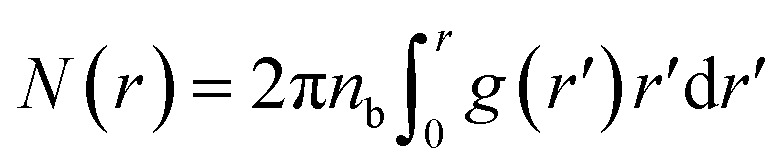
herein, *r* is an arbitrary distance from the center of the tagged nano-inclusion.

#### 3.1.1 Scaling theory predictions

As the density increases, the attractive forces begin to dominate^23,24^ and lead to a macroscopic aggregate of NIs. As a consequence, inclusions-density increases locally. In practice, a simple descriptive definition of the fractal dimension *d*_F_ is adopted,^55–58^ we consider a disc of radius *r* centred at some nano-inclusion taken as the origin of space, and we estimate the number of adjacent NIs inside it. Since the distribution of the NIs is random, the disc (or the aggregation space) is of fractal structure, with a fractal dimension denoted as *d*_F_. The latter is naturally less than the membrane surface dimension (*d* = 2). As a result, the number of NIs inside the disc that is directly proportional to their total mass identifies with the current coordination number, *N*(*r*), and we write the scaling relation,

13 *N*(*r*) = *r*^*d*_F_^

Using the definition of *N*(*r*) and its relation with RDF mentioned in the above section (eqn (12)) suggests that RDF behaves, as a function of the separation distance, as:14
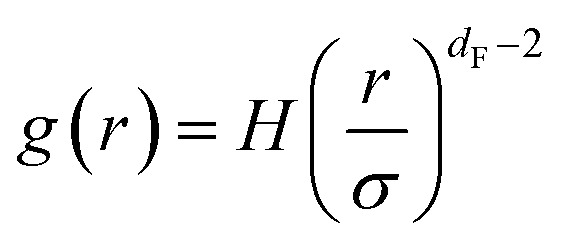
with *H* a positive dimensionless amplitude, *σ* = 2*R* accounts for the NIs diameter. Since *d*_F_ < 2, RDF decreases with distance, by the fact that the correlations between the NIs attenuate.

The first precious information we extracted from the RDF scaling-law deals with the principal peak height *g*_max_ and its width *r*_max_ variation as a function of the NIs number density. Such a law is also valid for *r* = *r*_max_, and we then write,15
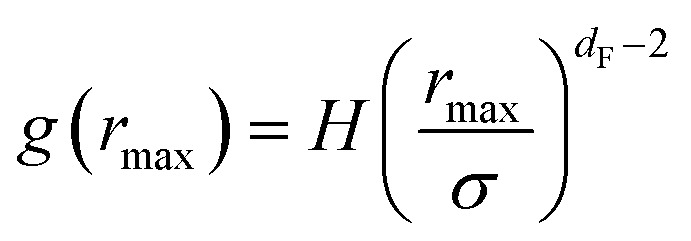
or in log–log scale,16
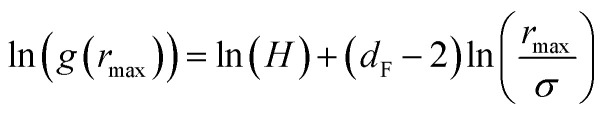
Then, the slope of the curve representing ln(*g*(*r*_max_)) as a function of ln(*r*_max_) makes it possible to determine the fractal dimension of the NIs aggregate, *d*_F_.

The Scaling Theory suggests that *d*_F_ does not depend on the number density *n*. Thus, the Scaling Theory predicts the *d*_F_ to be a universal quantity. The amplitude, *H*, can be obtained by extrapolation on the vertical axis. We recall that since, as part of the Scaling Theory, *d*_F_ < 2, *g*_max_ decreases with the position of the first peak of RDF, *r*_max_.

#### 3.1.2 Structure from MD simulation

**Fig. 2:**
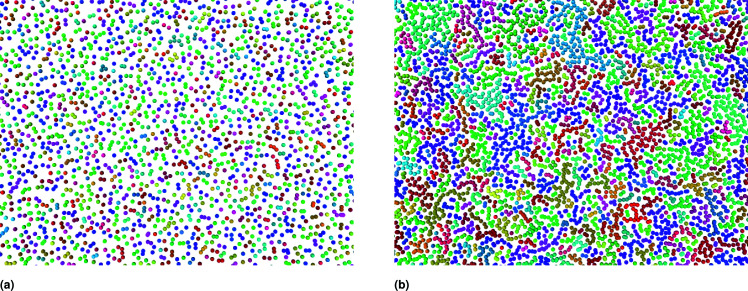
Slices from the square simulation box, captured for the characteristic number densities of NIs equilibrated at room temperature, using the Open Visualization Tool (OVITO) (https://www.ovito.org). (a) *n*_c_ = 3.67 × 10^−3^ Å^−2^, (b) *n*_g_ = 8.40 × 10^−3^ Å^−2^. To obtain the desired number density, the lengths of the simulation box were changed, but the NIs number remained fixed. The number of NIs used to produce the structural and dynamic properties is *N* = 10^6^. Different colors represent different clusters, which are an ensemble of connected particles. The particles belong to different clusters have no continuous path connecting them to a spanning network chains because of the weak correlation between them.

Fig. 2 depicts instantaneous snapshots at different number densities at *T* = 298.15 K. For low number density, more free-area is available, and the correlation between the NIs is weak. Analysis of the equilibrated particles configuration showed the apparition of small chains-like structures. Thus, these form small barriers and slightly limit the NIs diffusivity. At the critical number density *n*_c_, NIs form large aggregates manifesting as percolated gels (chains-like) and jammed clusters. Thus, each cluster gets stuck in a cage formed by its neighbouring clusters. In this case, the correlation between NIs is significant. Thereby, we expect slow dynamics. At the gelation number density *n*_g_, the NIs form crowded chains-like structures. In this case, the particles cannot move freely because of the dominant of the attractive interaction. Thus, as demonstrated for three dimensional^59,60^ and two dimensional colloidal systems dispersed in simple liquids,^61–65^ using computer simulations and experiments, we expect that NIs aggregate is of fractal dimension.

**Fig. 3:**
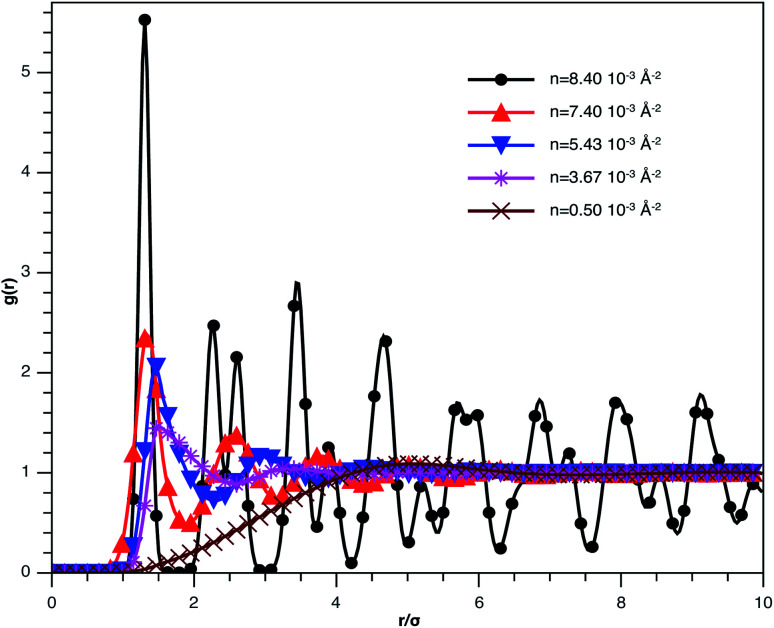
The radial distribution function *g*(*r*) between the BuSn12 NIs in the plane of the membrane bilayer, for different number densities, from MD simulation.

**Fig. 4:**
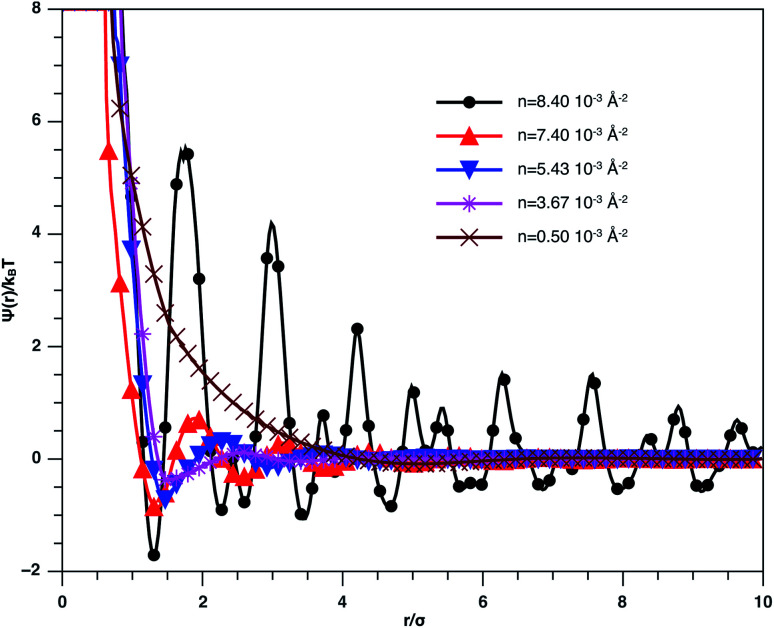
Mean effective interaction potential of the spherical NIs within the bilayers for different number densities, from MD simulation.

**Fig. 5:**
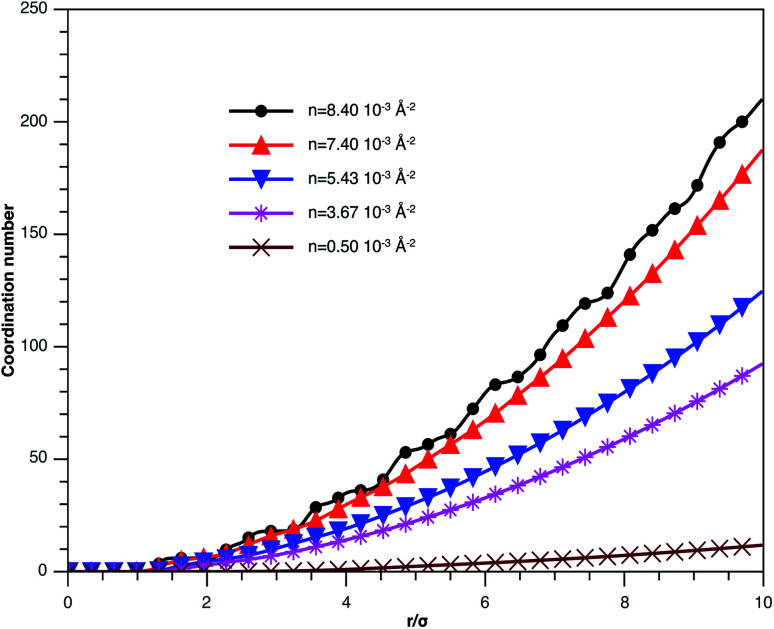
Coordination number *N*_c_ of the spherical NIs within the bilayers for different number densities, from MD simulation.

In Fig. 3, we report RDF *versus* the dimensionless distance, r* = *r*/*σ*, for several values of the NIs density, using MD simulation. Firstly, we remark that at a small distance, compared to the NIs diameter that is for 0 < *r* < *σ*, RDF practically vanishes due to the imposed strong steric repulsion between the adjacent NIs, which is applied to prevent the nanoparticles from overlapping. Then, for 0 < *r* < *σ*, the NIs present a correlation hole. For distances beyond *σ*, the NIs show a local order. The latter is due to a strong correlation between NIs. Of course, the size of this region crucially depends on the value of the physical parameters appearing in the interaction potential, in particular, the density of NIs. At a very high distance, however, RDF saturates naturally to 1, whatever is the value of density. This limit means that the NIs are completely disordered in space, *i.e.*, the order is absent at a long-range. Between the two distance-regimes, RDF oscillates and presents as a succession of peaks of decreasing heights that indicating the existence of many shells of neighbours. The locations of maxima of these oscillations correspond to the preferred relative distances between the neighbouring NIs. Secondly, as shown in Fig. 3, the position of the RDF first peak, *r*_max_, is shifted toward the left as the density of NIs increases. This shift is due to a net dominance of the attractive part of the mean-field interaction potential, which is related to the RDF by *g*(*r*) = exp(−*U*/*k*_B_*T*), as shown in Fig. 4. Then, the coordination number, *N*_c_, increases progressively, as observed in Fig. 5.

From Fig. 3, we extract the reduced maximum position of the principal peak, *r*_max_/*R*, for a wide range of NIs number density. A good fit with the calculated numerical data from MD simulation shows that *r*_max_ decreases with the NIs number density according to a straight line of the equation,17
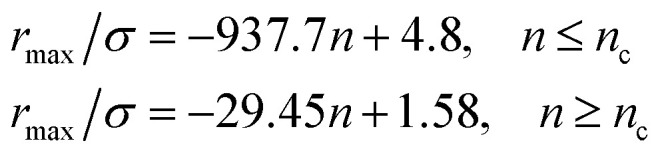


**Fig. 6:**
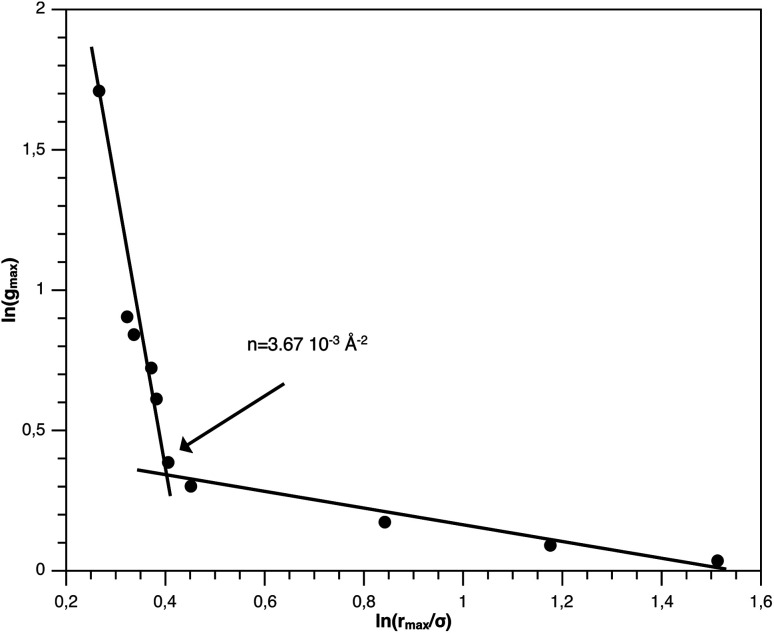
*g*_max_ as a function of *r*_max_ in logarithmic scale.

Also, Fig. 3 shows that the principal peak-height increased by an increase of the NIs density. We attribute such an increase to a progressive dominance of the attractive part of the mean-field interaction potential of Fig. 4. Thus, the latter gives rise to an aggregation of the NIs in the host membrane. The increase of the maximum, *g*_max_, as a function of *r*_max_/*σ* is depicted in Fig. 6. The best fit of numerical data gives the following form for *g*_max_.18
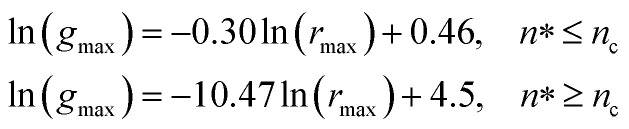


The MD calculation of *d*_F_ is in good agreement with the Scaling Theory, it suggests that for number densities below the gelation one *n* < *n*_c_, *d*_F_ does not depend on *n*. In this sense, it is a universal quantity. However, as we will see later, the Scaling theory fails to predict the *d*_F_ for number densities above the gelation density *n* > *n*_c_, giving negative value of *d*_F_. We add that the amplitude, *H*, can be obtained by extrapolation on the vertical axis. A comparison with the scaling-relation means that the NIs aggregate is fractal and we have, for *n* ≤ *n*_c_, *d*_F_ = 2 − 0.30 = 1.70. This value is slightly great than the fractal dimension calculated for simple colloidal systems, *d*_F_ = 5/3,^63^ that found in good agreement with other experimental results.^62,65^

These results inspire a more general understanding of the liquid–gel transition in colloidal systems. To discuss the possibility of a reverse gelation process, we recall that the production of amorphous solids from an ordered solid has been identified.^66,67^ In practice, this transition can be made under the effect of heating or stretching the materials,^68,69^ which will increase the volume and therefore decrease the density. The flow process, which thins an initial solid material, can be seen as a process opposite to gelation, which solidifies an initially liquid sample. It seems that the observed appearance of stiffness from a fluid state and the onset of the flow of a solid-state are two almost mirroring manifestations of the phase transition.^21^

### 3.2 Thermodynamic properties

The aim of this section, recalling that we conducted the simulations in fixed temperature, is to study the effect of NIs number density on the microscopic potential energy, *E*_pot_, and the pressure, Press.

We calculated the potential energy as:19
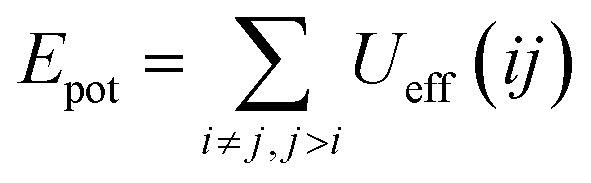
where *U*_eff_(*ij*) is the effective interaction potential defined above.

**Fig. 7:**
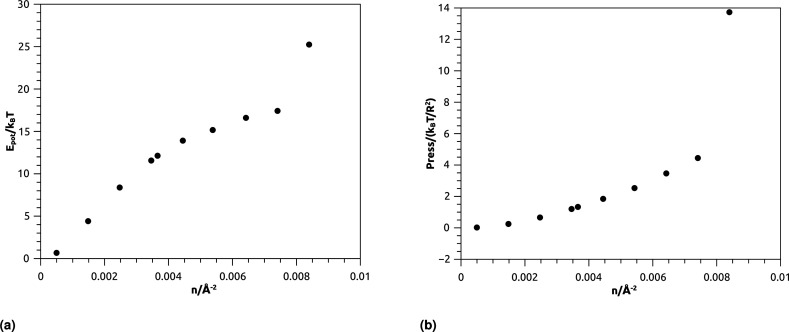
Thermodynamical properties from MD simulation as a function of nano-inclusion density: (a) potential energy, (b) pressure.

We calculated the pressure as:20
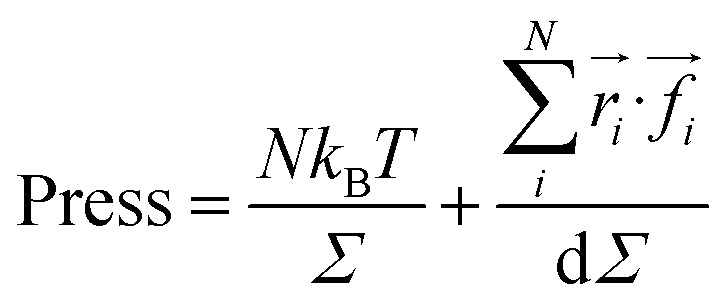
where the first term represents the kinetic pressure due to the NIs choc with the walls of the simulation square, like an ideal gaze, and the second term is the Virial Pressure, which is due to the inter-particles choc. Fig. 7a shows the variation of the potential energy (*E*_pot_) as a function of the NIs number density (*n*). We note that the *E*_pot_ is close to zero for very low densities but shows a logistic growth when (*n*) increases. For *n* ≤ *n*_g_, *E*_pot_ shows a rapid linear increase. For *n* > *n*_g_, *E*_pot_ shows a slow increase. But when the numerical density reaches the gelation density (*n* = *n*_g_), *E*_pot_ shows a sharp jump. The increase in *E*_pot_ is due to an increase in the correlation between particles, as quantitatively shown in Fig. 3–5.

Fig. 7b represents the variation of the pressure (Press) as a function of *n*. The pressure gradually increases with the numerical density, with a relatively faster increase for *n* > *n*_g_, and as well as *E*_pot_, shows a noticeable jump for *n* = *n*_c_. The numeric values of *E*_pot_ and Press are given in Table 1.

**Table 1:** Reduced values of potential energy and pressure as a function of NIs number density

*n*/(10^−3^ Å^−2^)	Potential energy/*Nk*_B_*T*	Pressure/(*k*_B_*T*/*R*^2^)
0.50	0.0268648	0.67979665
1.48	0.25397951	4.4172149
2.47	0.66446969	8.3937248
3.45	1.2060298	11.56985
3.67	1.3359001	12.137332
4.45	1.8514666	13.917294
5.43	2.53801	15.1734
6.42	3.4698504	16.610507
7.40	4.4472106	17.42904
8.40	13.734708	25.251927

### 3.3 Dynamic properties

#### 3.3.1 Theoretical prediction of dynamic properties for low density nano-inclusions

Nano-inclusions experience a ballistic diffusion, for early times, because of the absence of the correlations. At these small-time-scales, the random walker is not yet gets stuck in a cage. As we shall describe below, such a regime is put in evidence by our MD simulations, at the short times.

We describe the Brownian motion of a NIs by the following phenomenological Langevin equation:21
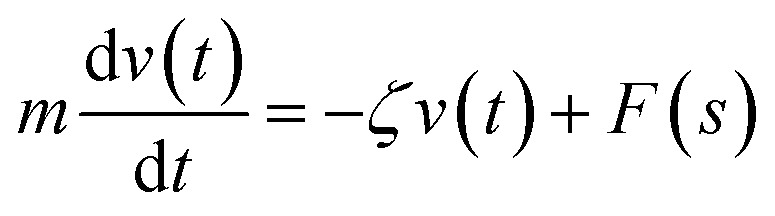
where *m* stands for the mass of the tracer, *v*(*t*) for its velocity, *ζ* for the friction coefficient, and *F*_s_(*t*) for a random force. The friction coefficient *ζ* is related to the viscosity of the NIs, *η* and the NIs radius, *R*, by the classical Stokes relation *ζ* = 6π*ηR*. Of course, the viscosity of the solution depends on the number density of the dispersed nanoparticles *n*. For small values of the number density, this effective viscosity obeys the well-known law,^70^22
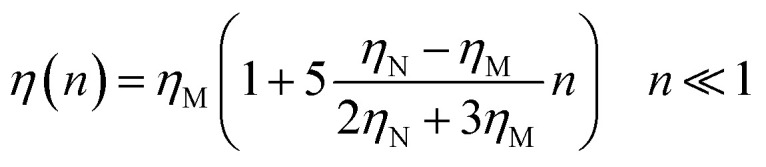
here *n* accounts for the reduced NIs number density, *η*_M_ is the viscosity of the membrane, and *η*_N_ is that of NIs. As it should be, we have *η*(*n*) > *η*_M_, since *η*_N_ > *η*_M_. We return to the stochastic force, and we see that we considered as white noise with:

23 〈*F*(*t*)〉 = 0

24 〈*F*_s_(*t*)·*F*_s_(0)〉 = 6*k*_B_*Tζδ*(*t*)

where *δ*(*t*) is the Dirac distribution. The brackets 〈…〉 mean an average over time.

In the following, we are interested in the mean-square-displacement (MSD) defined as 〈(*r* − *r*_0_)^2^〉 ≡ 〈*Δ*^2^*r*〉, such as:25
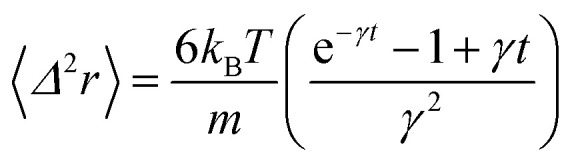
with *γ* = *ζ*/*m* knowing as the relaxation rate. For times much longer than the inverse relaxation rate, that is for *t* ≫ *γ*^−1^ ≡ *τ*, MSD grows linearly with time, and depend on the diffusion coefficient *D*, defined as the compromise between the thermal fluctuations and the dissipation such as 
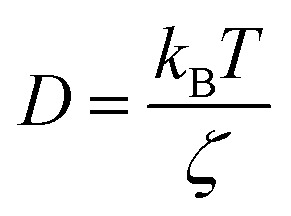
 in two dimensions, by:

26 〈*Δ*^2^*r*〉 = 4*Dt*, *t* ≫ *τ*

For *t* ≪ *τ* the Taylor development of e^−*γt*^ gives: 
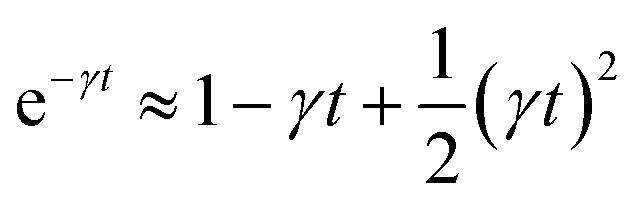
, then we find:27
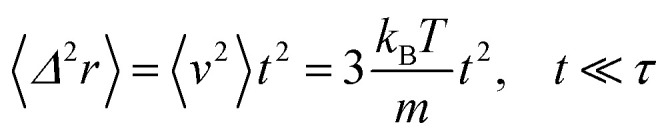


#### 3.3.2 Theoretical prediction of dynamic properties for high density nano-inclusions

When the number density of NIs is riched *n*_c_ their dynamics are more complex than those in low number density. In this case, NIs particles move ballistically at short times. Thus the mean-square displacement 〈*Δ*^2^*r*〉 ∼ *t*^2^, which is followed by a crossover to Fickian diffusion, characterized by 〈*Δ*^2^*r*〉 ∼ *t* for long times. But, in dense colloidal systems a caging effect, where the atoms are trapped by their neighbors, cause a subdiffusion regime that intermediate the ballistic and the diffusive regimes. This regime is characterized by 〈*Δ*^2^*r*〉 ∼ *t*^*α*^, with 0 ≤ *α* ≤ 1. *α* goes to a limit value when the number density reaches the gelation one that we note *n*_g_.

The analytical determination of the value of *α* for the subdiffusive regime remains uncertain. However, to predict an approached value of this key parameter, there are developed theoretical approaches based on the inclusion of complex phenomenological memory functions in the generalized Langevin equation. In this context, Zwanzig has developed a Dynamics Theory based on the generalized Langevin equation (GLE) instead of the standard Langevin equation (SLE).^71,72^ This approach can be adopted to discuss the subdiffusion observed in NIs, which we considered as two-dimensional colloidal particles swimming in a solvent (the host membrane). From a mathematical point of view, it is an extension of the SLE developed to study the dynamics behaviour of simple liquids, where the friction is assumed to be determined using the instantaneous velocity of the particles under study. We note that the difficulty lies in understanding the phenomena of subdiffusion is to explain the cage effect mathematically. Besides, physically speaking, the subdiffusion depends on various parameters, namely, temperature, number density, and finally, and least lost the nature of the host medium. In this context, we express the GLE as,28
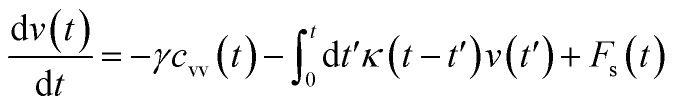
where *v* the velocity of a moving nano-inclusion (a tracer). 
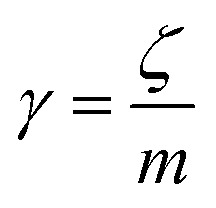
 is the relaxation rate, where *ζ* is the friction coefficient, and *m* is the mass of the tracer. *κ* is the memory-function that expresses the friction retardation, and *F*_s_(*t*) is a random force felt by the moving nano-inclusion due to its collisions with the molecules of the host membrane. Thus, the random force verify the equation,

29 〈*F*_s_(*t*)·*F*_s_(0)〉 = 6*mk*_B_*T*[*γδ*(*t*) + *κ*(*t*)], *t* > 0

For these considerations, VACF solves the following differential equation:30
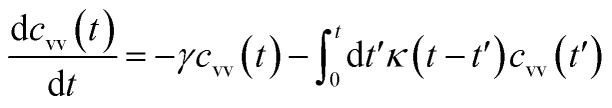


For the analytical resolution of the GLE equation, we refer to an elegant memory function, proposed by Flenner *et al.*,^73^ originally developed to study the dynamics aspect of lipid atoms, which also presents a subdiffusion phenomenon. This approach base on the Zwanzig–Mori projection method to model the 〈*Δ*^2^*r*〉 behaviour.

This is done by involving the equation of motion for the density autocorrelation function *Φ*_s_(*q*, *t*) = 〈*n*(−*q*, 0)*n*(*q*, *t*)〉 of a tracer, *i.e.* a selected particle, at the wave vector *q*,^74^31
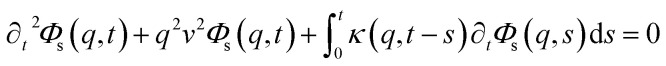
where 
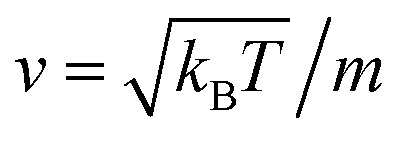
, *k*_B_ stand for the Boltzmann's constant, *T* denote the temperature and *m* refer to the nano-inclusion mass.

We obtain the two-dimensional equation of motion of the MSD from:32
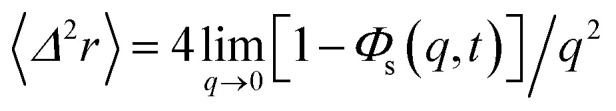
which gives,33
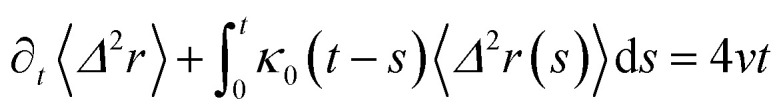
where 
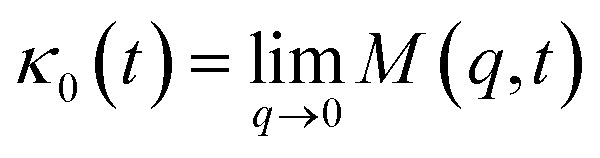
.

The Flenner *et al.* approach consists of introducing an adequate memory function that explicit three crossover times scales and thus reproduce the ballistic, the normal, and the subdiffusive regimes,^73^34
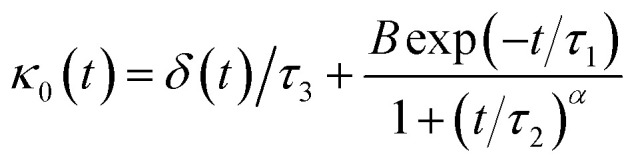
where *δ*(*t*) is the Dirac delta function, *B* is a dimensionless parameter. *τ*_1_ is the characteristic time for the crossover from the subdiffusion to the normal diffusion. *τ*_2_ is the onset time of the subdiffusion regime. And, *τ*_3_ is a characteristic time for the crossover from the ballistic to the subdiffusive regime.

We note that, for *B* = 0, we recover the standard Brownian diffusion laws, and the 〈*Δ*^2^*r*〉 behaves like:35
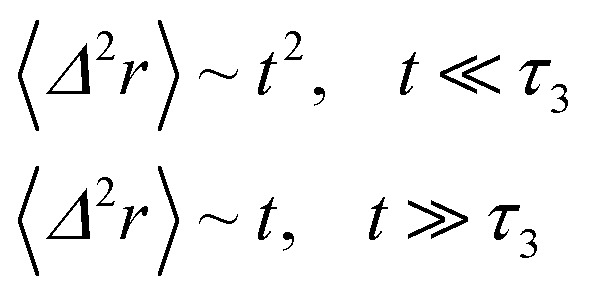


If (*t*/*τ*_2_)^*α*^ ≪ 1, then36
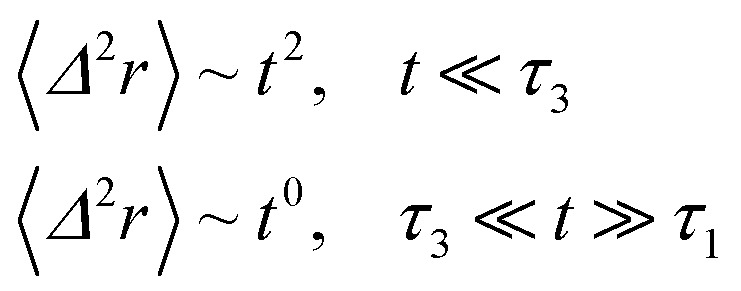


The power-law term of the memory function represents the subdiffusion phenomenon. Flenner *et al.* performed a numerical computation, taking into account this term,^73^ and revealed that there is a subdiffusive regime with *α* < 1 intermediating the ballistic and normal regimes. This region is due to a cage effect applied to a considered particle by its surrounding neighbors. Thus, the mean-square-displacement, 〈*Δ*^2^*r*〉, is expressed as:

37 〈*Δ*^2^*r*〉 ∼ *t*^*α*^ (*α* < 0), *t* > *τ*_2_

#### 3.3.3 Analysis of MD simulation results

In the last years, MD simulation becomes a privileged method for the statistical description of dynamic properties. In the framework of this method, the dynamics behavior is characterized quantitatively by the mean-square-displacement (MSD), the velocity autocorrelation-function (VACF), and the diffusion coefficient (*D*_*α*_).

In this section, we shall discuss the influence of the number density variation on the dynamic properties of the BuSn12 membrane NIs, using *NΣT*-MD simulations. We recall that the considered NIs are characterized by a radius *R* = 4.5 Å and molecular weight of 2866.7, the temperature fixes to room temperature, but the number density of NIs varies on a wide range.

**Fig. 8:**
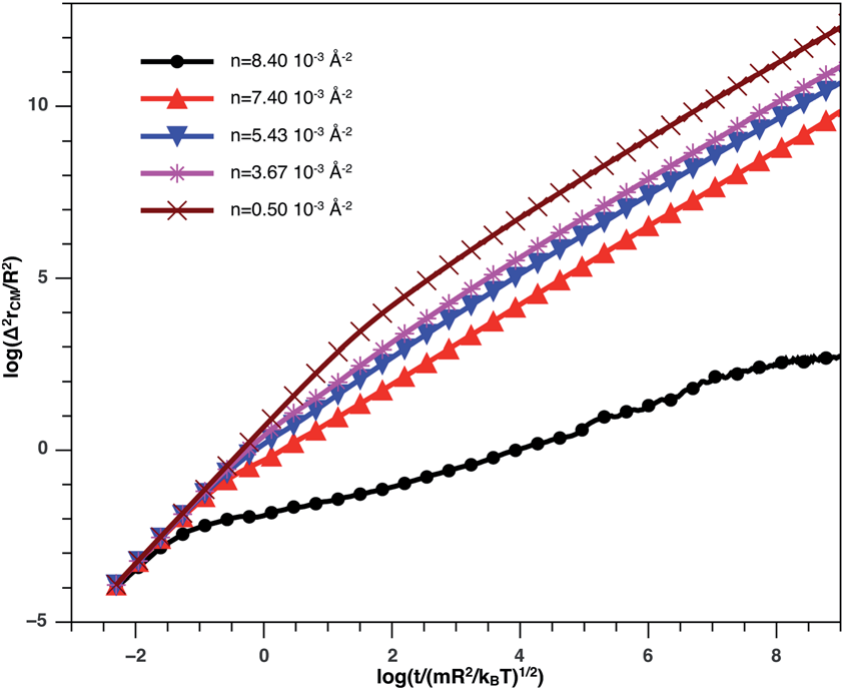
log–log plot of the MSD *versus* time for the nano-inclusion particles within the bilayers, we calculated from MD simulation.

In Fig. 8, we present the log–log curve of MSD as a function of reduced time, for different nano-inclusion number density values. For low densities (*n* < *n*_c_ = 3.67 × 10^−3^ Å^−2^), we note that the MSD have two diffusion regimes characterized by a transition time (*t* ≤ *τ*), *i.e.* a ballistic diffusion with an exponent (*α* = 2) for *t* ≤ *τ* and a normal diffusion (*α* = 1) for *t* > *τ*, we notice that *τ* decreases when *n* increases, that is to say that the diffusion of the nano-inclusion quickly reaches the normal diffusion. Moreover, for high densities *n* > *n*_c_, we note that the MSDs curves have three diffusion regimes. At more important times, there is a crossing towards the Fickian diffusion with 〈*Δ*^2^*r*〉 = 4*D*_*α*=1_*t*, where *D*_*α*=1_ is the long time self-diffusion coefficient. But, in this crossing region, from the ballistic to the normal diffusion, a subdiffusive region appears where 〈*Δ*^2^*r*〉 = 4*D*_*α*_*t*^*α*^, with *α* < 1. The value of *D*_*α*<1_ is smaller for high density and has a limit value of about 0.5 for the first gelation number density (*n*_g_ = 8.40 × 10^−3^ Å^−2^).

**Fig. 9:**
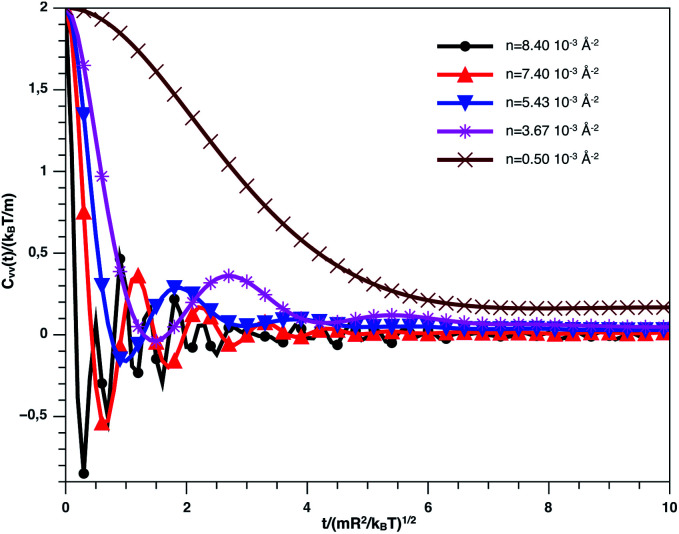
VACF *versus* time for the nano-inclusion particles within the bilayers from MD simulation.

Fig. 9 describes the variation of VACF as a function of time for several number densities, below and above *n*_c_. We characterize this function by more pronounced damping oscillations at high density. This behavior is in good agreement with the results of the theory described above. In high density, we attribute the VACF oscillations, which take negative values, to the confinement of each nano-inclusion in a cage formed by their close neighbors. These oscillations signify that the diffusion has a memory. On the other hand, VACF is positive and has few damping oscillations for low densities as a comparison to the high-density systems. Also, we note that the asymptotic behavior of VACF, which reflects the diffusion regime of a studied system, tends towards zero negatively for high-density values, according to the theory described above.

In Table 2, we give the diffusion exponent, *α*, and the corresponding crossovers time for *n* < *n*_c_. We note that *α* takes two values, 2 and 1, corresponding to the ballistic and normal regimes, respectively. The crossover time from the ballistic regime to the normal one decrease as the number density increase. This decrease is due to an increase in the correlation between NIs.

**Table 2:** Diffusion exponent *α* and the correspond crossover times for *n* < *n*_g_

*n* (10^−3^ Å^−2^)	Time interval	*α*
0.50	*t* ≤ 1.13 × 10^−10^ s	2
	*t* > 1.13 × 10^−10^ s	1
1.48	*t* ≤ 8.34 × 10^−11^ s	2
	*t* > 8.34 × 10^−11^ s	1
2.47	*t* ≤ 5.06 × 10^−11^ s	2
	*t* > 5.06 × 10^−11^ s	1
3.45	*t* ≤ 3.75 × 10^−11^ s	2
	*t* > 3.75 × 10^−11^ s	1

In Table 3, we give the diffusion exponent, *α*, and the corresponding crossovers time for *n* ≥ *n*_c_. We note that *α* takes three values, namely, the two values observed for *n* < *n*_c_ (2 and 1), in addition to the value *α* < 1 corresponding to a subdiffusive regime, which intermediates the ballistic and normal regimes, this is due to the cage effect discussed above. We note that, for the subdiffusive regime, *α* is not unique for all densities. In fact, *α* decrease progressively and reaches a limit value *α* = 0.5 for *n*_g_.

**Table 3:** Diffusion exponent *α* and the correspond crossover times for *n* ≥ *n*_c_

*n* (10^−3^ Å^−2^)	Time interval	*α*
3.67	*t* < 1.94 × 10^−11^ s	2
	1.94 × 10^−11^ s ≤ *t* ≤ 4.81 × 10^−11^ s	0.98
	*t* > 4.81 × 10^−11^ s	1
4.45	*t* ≤ 1.68 × 10^−11^ s	2
	1.68 × 10^−11^ s ≤ *t* ≤ 5.06 × 10^−11^ s	0.94
	*t* > 5.06 × 10^−11^ s	1
5.43	*t* ≤ 1.52 × 10^−11^ s	2
	1.52 × 10^−11^ s ≤ *t* ≤ 5.21 × 10^−11^ s	0.91
	*t* > 5.21 × 10^−11^ s	1
6.42	*t* ≤ 9.24 × 10^−11^ s	2
	9.24 × 10^−11^ s ≤ *t* ≤ 3.07 × 10^−11^ s	0.86
	*t* > 3.07 × 10^−11^ s	1
7.40	*t* ≤ 9.06 × 10^−12^ s	2
	9.06 × 10^−12^ s ≤ *t* ≤ 3.39 × 10^−11^	0.80
	*t* > 3.39 × 10^−11^	1
8.40	*t* ≤ 2.65 × 10^−12^	2
	*t* > 3.95 × 10^−12^	0.5

**Fig. 10:**
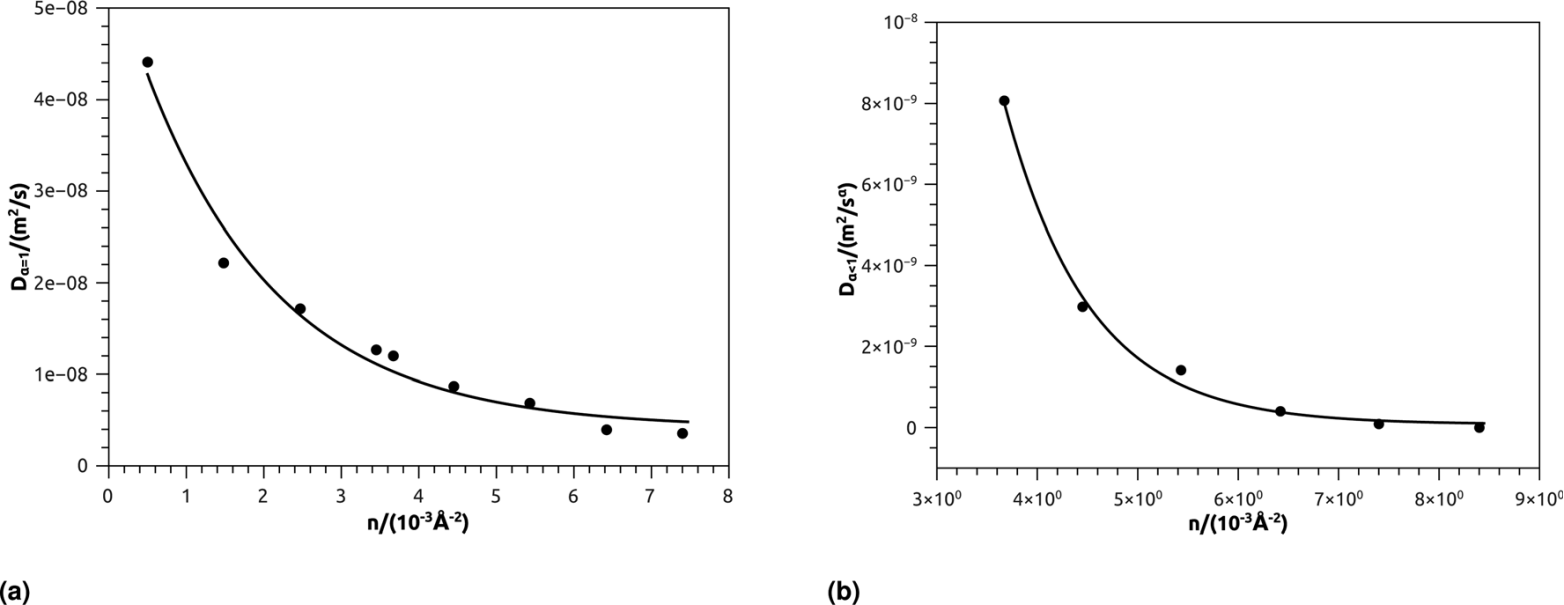
(a) The normal diffusion coefficients *D*_*α*=1_, and (b) the subdiffusive diffusion coefficients *D*_*α*<1_ as a function of the number density *n*, the solid line depict the best exponential decay fit.

In Table 4, we give the normal diffusion coefficient of BuSn12 NIs, *D*_*α*=1_, in reduced units and SI units, as a function of *n*. In Table 5, we give the subdiffusion coefficient *D*_*α*<1_, corresponding to the subdiffusive regime. We note that the subdiffusion appears for a small time-interval except for the gelation number density *n*_g_ the NIs dynamics is blocked for a long time. Fig. 10a and b depict the diffusion coefficients *D*_*α*=1_ and *D*_*α*<1_, for the BuSn12 NIs, giving in Tables 4 and 5, respectively.

**Table 4:** Normal diffusion coefficient *D*_*α*=1_

*n* (10^−3^ Å^−2^)	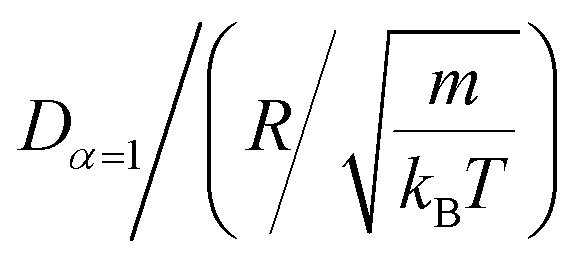 a	*D*_*α*=1_ b/10^−8^ m^2^ s^−1^
0.5	3.34	4.41
1.48	1.68	2.22
2.47	1.3	1.72
3.45	0.96	1.27
3.67	0.91	1.20
4.45	0.66	0.871
5.43	0.52	0.686
6.42	0.3	0.396
7.40	0.27	0.356

a
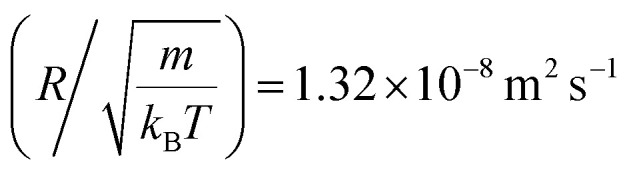
, for BuSn12 nano-inclusions.

b *D*_*α*=1_ for BuSn12 nano-inclusions.

**Table 5:** Generalized diffusion coefficient *D*_*α*<1_

*n* (10^−3^ Å^−2^)	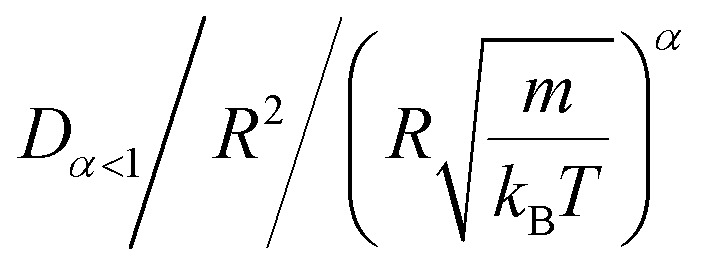 a	*D*_*α*<1_ b/10^−9^ m^2^ s^−*α*^
3.67	0.48	8.07
4.45	0.37	2.98
5.43	0.29	1.41
6.42	0.22	4.06 × 10^−1^
7.40	0.17	9.12 × 10^−2^
8.40	0.11	5.19 × 10^−5^

a
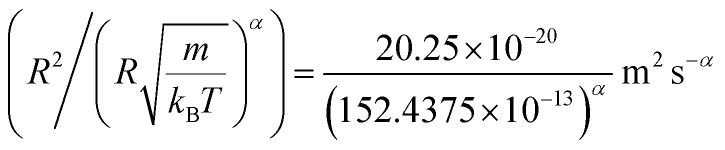
, for BuSn12 nano-inclusions.

b *D*_*α<*1_ for BuSn12 nano-inclusions.

We report that the variation of the *D*_*α*=1_ and *D*_*α*<1_ as a function of the number density *n* follow a first-order exponential decay (Arrhenius-like decay).

For *D*_*α*=1_ we have,

38 *D*_*α*=1_ = *D*_0_ + *D*_1_e^−*n*/*n*_0_^

with the best fitting parameters

39 *D*_0_ = 4.16 × 10^−9^ m^2^ s^−1^ ± 1.67 × 10^−9^ m^2^ s^−1^

40 *D*_1_ = 5.16 × 10^−8^ m^2^ s^−1^ ± 0.35 × 10^−9^ m^2^ s^−1^

41 *n*_0_ = 1.73 × 10^−3^ Å^−2^ ± 0.27 × 10^−3^ Å^−2^

and for *D*_*α*<1_ we have,

42 *D*_*α*=1_ = *D*_0_ + *D*_1_e^−*n*/*n*_g_^

with the best fitting parameters

43 *D*_0_ = 7.96 × 10^−11^ m^2^ s^−1^ ± 1.77 × 10^−10^ m^2^ s^−1^

44 *D*_1_ = 6.25 × 10^−7^ m^2^ s^−1^ ± 2.93 × 10^−7^ m^2^ s^−1^

45 *n*_g_ = 8.4 × 10^−3^ Å^−2^ ± 8.50 × 10^−5^ Å^−2^

We note certain limitations of this study. First, although the calculated properties follow well-established physical laws, the fitted parameters are not universal and depend on other relevant factors, such as the shape and size of the NIs. Therefore, projecting the results of the present study on non-spherical NIs may be inappropriate. Second, we also planned to explore the physical properties at *n* > *n*_g_, but the MD simulation code is proving to be unstable. Possibly the MD divergence is due to the attractive part of the interaction potential.

From the literature, for colloidal suspensions in simple solvent (water, for example), it is known that the subdiffusion phenomenon occurs at high enough number density, which induces the cage effect,^75,76^ where the diffusion of a selected particle getting trapped in a cage formed by its close neighbors. In contrast, in the present case, we mark that this phenomenon appears for low number densities. It is natural to link the membrane NIs slow diffusion to the nature of the host medium. The latter is a biological membrane composed of highly ordered lipids that tend to limit their diffusivity.

To compare the diffusion of proteins and the diffusion of inorganic NIs within biological membranes, we refer to our recent work.^36^ In this previous work, we have studied the subdiffusion of cylindrical shaped proteins inclusion in phospholipids mixture bilayer, using coarse-grained MD simulations. Accordingly, for the room temperature, we found a mean subdiffusion exponent of proteins to be *α* = 0.65, and we found the diffusion coefficient of the order of 10^−16^ m^2^ s^−1^ this range is also found in some experimental works.^77–79^

In the present work, the diffusion of inorganic nano-inclusion is in the range of 10^−14^ to 10^−9^ m^2^ s^−1^. These values overlap with those of other similar works, which is in the order of 10^−14^ m^2^ s^−1^,^80,81^ performed for a number density close to *n*_g_.

Thus, we deduce that the diffusion of inorganic NIs is faster in comparison to the organic inclusions.

We recall that synthetic membranes can be composed of single lipids type, the liposome membranes as an example.^82,83^ But the biological membranes are crowded structures encompassing several lipids types, included and external proteins, and present in different phases, the pulmonary surfactant as an example.^36^ Thus, the expensive details related to the membrane nature limit the studied number of NIs in an MD simulation. For this purpose, a direct interaction potential between NIs, which respect the membrane fluctuation, permits the study of a large NIs number. Thus, an effective interaction potential cannot provide nanoscopic properties. Although, it gives an overview of the NIs mesoscopic properties. In this context, the NIs are considered a two-dimensional colloidal dispersion system. For simplicity, the nano-inclusions represent the colloidal dispersion, and the membrane is the two-dimensional solvent.

In this work, we studied the BuSn12 nanoparticles as membrane nano-inclusions, and we assumed that they are hard-spheres and charge neutral. We perceive that their interaction-potential form with the appropriate parameters is determined experimentally in previous works by D. Constantin.^37^

Generally, nano-inclusions can be charged or neutral and present in different shapes and sizes, depending on their applications.^84–87^ From a mesoscopic viewpoint, MD simulations require appropriate interaction potential forms and parameterizations. These proposed models are often semi-empirical. For instance, the electrostatic interaction due to the surface charge can be modeled using a Yukawa screened coulombic interaction type, mimicking the standard DLVO model for charged colloidal particles in a simple solvent.^76,88^ Besides, the membrane fluctuation can also depend on the nano-inclusions shape. Thus, the interaction potential form and parameters are specific to each nano-inclusion type and cannot be precisely a Gaussian type.

Consequently, the three scientific approaches encompassing theory, experiment, and simulation are needed to explore the biophysical-chemistry prospects of various nano-inclusions types at different time-length scales.

## 4 Conclusion

In this work, we performed MD simulations in the *NΣT* statistical ensemble conditions to study membrane NIs, in which a specific type of nano-inclusion, BuSn12, is discussed in detail. We conducted the study for a wide range of NIs densities. As hypothesized, the calculated physical properties have shown a remarkable discontinuity, corresponding to the critical and gelation number densities. Thus we have provided a mathematical understanding of the spherical NIs aggregation.

While previous works study the interactions between BuSn12 NIs across the membrane, this study offers further physical insights into their phase transition phenomena. Accordingly, we discussed the structural properties relative to the number density by developing a Scaling Theory approach. This approach base on the idea that the NIs aggregate is of fractal structure. Such a scaling method consists with the MD simulation for *n* < *n*_c_ but diverge for *n* > *n*_c_. We determined the thermodynamic properties through the potential energy per nano-inclusion and the pressure, using MD simulation. Thus, we observe that these major physical quantities present a discontinuity for *n* = *n*_c_ and a clear jump for *n* = *n*_g_. We studied the dynamic properties and their dependence on the NIs number density through the temporal evolution of the mean-square-displacement, the velocity auto-correlation-function, and the diffusion coefficients. Precisely, we explore the cage effect and the subdiffusion phenomenon in membrane NIs, using two approaches, which are a Dynamics Theory, based on the generalized Langevin equation, with the Flenner memory function, and MD simulation. We observe that the two dynamics approaches are in good agreement. Thus, the thermodynamics and dynamic properties analysis allow determining *n*_c_ and *n*_g_.

Gelation is a common phenomenon in colloidal science, which has several nanotechnological and medical implications. Thus, the simulation protocol we performed to study spherical membrane NIs, using a solvent-free interaction potential, can be adapted to different shapes and types of NIs, such as nano-rods and nano-discs. The approaches we used to determine the phase transition parameters can be conducted for other colloidal systems, such as microemulsions and metallic nanoparticles. In particular, to reveal drug delivery and cancer treatment.

## Conflicts of interest

There are no conflicts to declare.

## Supplementary Material
